# Crystal structures of β-carboxysome shell protein CcmP: ligand binding correlates with the closed or open central pore

**DOI:** 10.1093/jxb/erx070

**Published:** 2017-03-28

**Authors:** Anna M Larsson, Dirk Hasse, Karin Valegård, Inger Andersson

**Affiliations:** Laboratory of Molecular Biophysics, Department of Cell and Molecular Biology, Uppsala University, Husargatan, Uppsala, Sweden

**Keywords:** BMC domain, carboxysome, β-cyanobacteria, gated transport, microcompartment, shell protein

## Abstract

Cyanobacterial CO_2_ fixation is promoted by encapsulating and co-localizing the CO_2_-fixing enzymes within a protein shell, the carboxysome. A key feature of the carboxysome is its ability to control selectively the flux of metabolites in and out of the shell. The β-carboxysome shell protein CcmP has been shown to form a double layer of pseudohexamers with a relatively large central pore (~13 Å diameter), which may allow passage of larger metabolites such as the substrate for CO_2_ fixation, ribulose 1,5-bisphosphate, through the shell. Here we describe two crystal structures, at 1.45 Å and 1.65 Å resolution, of CcmP from *Synechococcus elongatus* PCC7942 (*Se*CcmP). The central pore of CcmP is open or closed at its ends, depending on the conformation of two conserved residues, Glu69 and Arg70. The presence of glycerol resulted in a pore that is open at one end and closed at the opposite end. When glycerol was omitted, both ends of the barrel became closed. A binding pocket at the interior of the barrel featured residual density with distinct differences in size and shape depending on the conformation, open or closed, of the central pore of *Se*CcmP, suggestive of a metabolite-driven mechanism for the gating of the pore.

## Teaser

A correlation between the conformation of the gating residues and the size and shape of the bound compound suggests a metabolite-driven mechanism for transport across the carboxysome shell.

## Introduction

Carboxysomes are bacterial protein-bound microcompartments with the function to increase the concentration of carbon dioxide (CO_2_) close to the carbon-fixing enzyme Rubisco (ribulose-1,5-bisphosphate carboxylase/oxygenase). CO_2_ is assimilated by the Rubisco-catalysed carboxylation of ribulose 1,5-bisphosphate (RuBP) to produce two molecules of 3-phosphoglycerate (3PGA). Rubisco is characterized by a low carboxylation turnover rate and is further slowed by a competing oxygenase activity. Cyanobacteria are highly productive organisms on a global scale (Field *et al.*, 1998), but their efficiency is impeded by slow CO_2_ diffusion and a low CO_2_ concentration in aqueous environments. To overcome this crippling condition, cyanobacteria and some chemoautotrophic bacteria have evolved carboxysomes, which encapsulate Rubisco and carbonic anhydrase in a semi-permeable icosahedral protein shell. HCO_3_^−^ is actively pumped into the cell and enters the carboxysome where it is converted to CO_2_ by the activity of carbonic anhydrase (reviewed in Price *et al.*, 2008). Positively charged channels in the protein shell are believed to allow entry of the charged HCO_3_^−^ molecule into the carboxysome but to prevent diffusion of CO_2_ back into the cytosol, thereby creating an environment with an elevated CO_2_ concentration near Rubisco (Dou *et al.*, 2008).

Two different types of carboxysomes occur, named α and β, based on gene arrangement and the form of encapsulated Rubisco (reviewed in Rae *et al.*, 2013). In α-carboxysomes, the *cso* operon codes for Form 1A Rubisco, shell proteins, β-carbonic anhydrase, and a protein suggested to have an essential role in α-carboxysome assembly (CsoS2; Cai *et al.*, 2015). β-Carboxysomes instead contain Form1B Rubisco and a γ-carbonic anhydrase analogue, and their shell proteins are encoded by the more scattered *ccm* genes (Tanaka *et al.*, 2008). The shell proteins in the two types of carboxysomes are orthologues and share a common fold. In the present models of bacterial microcompartments (Tanaka *et al.*, 2008), the facets are built up by several paralogues containing one or two copies of the bacterial microcompartment (BMC) domain (PF00936; Punta *et al.*, 2012) forming hexamers, or pseudohexamers in the case of the tandem BMC domains (Cai *et al.*, 2013). The less abundant pentamer-forming proteins (PF03319) are believed to close the vertices of the icosahedral compartment (Tanaka *et al.*, 2008; Wheatley *et al.*, 2013) and prevent diffusion of CO_2_ from the carboxysome (Cai *et al.*, 2009). Analysis of knockouts of the *ccm* shell proteins in *Synechococcus elongatus* PCC 7942 show that inactivation of the genes coding for the most abundant shell proteins, CcmK2, the tandem BMC domain protein CcmO, or the pentameric vertex protein CcmL, generate high-CO_2_-requiring mutants (Rae *et al.*, 2012). The double inactivation mutation of *ccmK3* and *ccmK4* resulted in normal appearance of carboxysomes but functional deficiency under low CO_2_ conditions (Rae *et al.*, 2012).

CcmP is a less abundant and recently identified shell protein (Cai *et al.*, 2012, 2013) that comprises two BMC domains. The overall fold of CcmP is similar to that of the other facet shell protein, CcmK, but the connectivity between the secondary structure elements varies due to rearrangements in the genome that result in a circularly permuted protein (Crowley *et al.*, 2008). The two-domain CcmP forms a trimer resembling the hexamer of the CcmK proteins. In addition, two trimeric CcmP rings appear to be stacked on top of each other and form a barrel (Cai *et al.*, 2013). Three-dimensional crystal structures of CcmP (Cai *et al.*, 2013) and its orthologue in α-carboxysomes, CsoS1D (Klein *et al.*, 2009), show that the pores at the three-fold symmetry axis may be either open or closed. The open central pore (~13 Å diameter) of these permuted tandem shell proteins is wider than those of the CcmK proteins (reviewed in Yeates *et al.*, 2011, 2013; Kinney *et al.*, 2011) and may potentially allow larger metabolites, such as RuBP and 3PGA, to pass through. The alternative conformations were suggested to facilitate the selective transport of metabolites through the protein shell (Klein *et al.*, 2009; Cai *et al.*, 2013). However, specific details on the transport process, in terms of the identity of the metabolites transported or the interactions that might trigger the conformational changes leading to the shuttling of metabolites, are presently lacking.

Here we describe two high-resolution structures of CcmP from *S. elongatus* PCC 7942 (named *Se*CcmP_*P*2_1_3 and *Se*CcmP_*I*2_1_3) co-crystallized with RuBP, the substrate of Rubisco. *Se*CcmP_*P*2_1_3 crystals were obtained from a protein solution containing glycerol, whereas *Se*CcmP_*I*2_1_3 crystals were obtained from protein produced free of glycerol throughout, including protein expression, purification, and crystallization. Both structures consist of a barrel formed by two layers of a *Se*CcmP trimer with a central pore, but the two structures show distinct conformational states. In *Se*CcmP_*P*2_1_3, the pore is open at one end and closed at the opposite end, whereas in *Se*CcmP_*I*2_1_3, the pore is closed at both ends. Electron density for a bound ligand is found in a binding pocket which is located between the two BMC domains within the CcmP monomer.

## Materials and methods

### DNA cloning

The sequence of the gene (Synpcc7942_0520; UniProt id Q31QW7) coding for CcmP was amplified from *S. elongatus* PCC 7942 chromosomal DNA using Phusion High-Fidelity DNA PCR Master Mix (Thermo Scientific) with forward primer 5'- AGCGGCTCTTCAATGGGCGTTGAGCTGCGCAGT-3' and reverse primer 5'- AGCGGCTCTTCTCCCCTCCCGCGAGCGA TCGC-3'. The gene was cloned into pENTRY-IBA51 using a StarGate^®^ Combi Entry Cloning Set (IBA Life Sciences) and, after confirming the correct sequence, subsequently cloned into pPSG-IBA33 using Stargate^®^ Transfer Reagent Set (IBA Life Sciences), resulting in plasmid pPSG-IBA33_0520.

### Protein expression

pPSG-IBA33_0520 was transformed into *Escherichia coli* strain BL21-AI (Life Technologies) for heterologous protein expression. *Escherichia coli* was grown in 2× YT medium (1.6% tryptone, 1% yeast extract, 0.5% NaCl) containing 100 μg ml^−1^ ampicillin until OD_600_=0.7 was reached. Cells were cooled in an ice bath, and protein expression was induced by adding 0.02% l-arabinose. Cells were harvested after further cultivation for 16 h at 291 K.

### Protein purification

The *Se*CcmP protein was purified using a three-step protocol comprising affinity, ion exchange, and size exclusion chromatographies at room temperature using an NGC purifier system (BioRad). Cells were resuspended in binding buffer (20 mM Bis-Tris propane pH 8, 500 mM NaCl, 20 mM imidazole, 10% glycerol) and lysed by sonication. After clearing the lysate using centrifugation at 40 000 *g* (JA-25.50, Beckman Coulter), the supernatant was applied to a HisTrap™ HP column (5 ml, GE Healthcare), washed thoroughly with washing buffer (20 mM Bis-Tris propane pH 8, 500 mM NaCl, 65 mM imidazole, 10% glycerol), and eluted with a gradient of 65–500 mM imidazole in the same buffer. After lowering the salt concentration by diluting the eluate in buffer A (20 mM Bis-Tris propane pH 9.5, 10% glycerol), the sample was applied to a MonoQ column (GE Healthcare) and eluted with a 0–50% gradient of buffer B (20 mM Bis-Tris propane pH 9.5, 1 M NaCl, 10% glycerol). Size exclusion chromatography was carried out using a HiLoad 26/60 Superdex 200 column (GE Healthcare), equilibrated with buffer C (20 mM Bis-Tris propane pH 8, 100 mM NaCl, 10% glycerol) and a flow rate of 2 ml min^−1^. Glycerol, which was initially present in all buffers as a stabilizing agent, was found to bind to the protein (see below). In subsequent rounds of purification, glycerol was therefore excluded from all steps in the purification protocol. The purified *Se*CcmP was concentrated to a final concentration of ~3 mg ml^−1^ (Vivaspin, Sartorius Stedim Biotech).

### Protein crystallization and data collection

*Se*CcmP (3 mg ml^−1^ in 20 mM Bis-Tris propane, 50 mM NaCl, 10 mM glycerol) was co-crystallized with 50 mM RuBP at room temperature (293 K). Crystallization conditions were screened using commercial screens and a mosquito^®^ Crystal robot (TTP Labtech) with 0.2 μl drop size. The best crystals were obtained in 0.2 M Li sulphate, 0.1 mM Tris–HCl pH 7.5, and 5% (w/v) polyethylene glycol (PEG) 4000 (Proplex Screen, Molecular Dimensions). These crystals belong to space group *P*2_1_3 with unit cell parameters *a*, *b*, *c*=107.3 Å, α, β, γ=90°, and two molecules per asymmetric unit, giving a *V*_M_ of 2.1 Å^3^ Da^−1^ and 42% solvent content. Glycerol-free *Se*CcmP (3 mg ml^−1^ in 20 mM Bis-Tris propane, 50 mM NaCl) was crystallized at 293 K using the hanging drop vapour diffusion method. Crystals were obtained using a reservoir solution consisting of 0.1 mM Tris–HCl pH 7.0, 0.2 mM potassium thiocyanate, and 6% poly-γ-glutamic acid (Molecular Dimensions), with drops formed from 4 μl of protein solution, 4 μl of reservoir solution, and 1 μl of 1 M RuBP. Crystals appeared after a few days and belong to space group *I*2_1_3 with unit cell parameters *a*, *b*, *c*=178.7 Å, α, β, γ=90°, and three molecules per asymmetric unit. Prior to data collection, crystals were soaked in a reservoir solution containing cryoprotectant (20% glycerol), transferred into a nylon loop (Hampton Research), flash-cooled in liquid nitrogen, and maintained at 100 K for data collection. When glycerol was omitted, 25% ethylene glycol was used as cryoprotectant.

X-ray diffraction data were collected at 100 K on a Pilatus 6M-F detector at beam line ID29 of the European Synchrotron Radiation Facility (ESRF) in Grenoble, France.

### Structure determination and refinement

Diffraction data were processed using XDS (Kabsch, 2010) and scaled with AIMLESS (Evans, 2006). Phases were obtained by molecular replacement in PHASER (McCoy *et al.*, 2007) using one monomer of the *Se*CcmP X-ray crystal structure (PDB code 4HT5) as a search model for the *Se*CcmP_*P*2_1_3 and a single BMC domain for the *Se*CcmP_*I*2_1_3 structure. ZANUDA in the CCP4 suite (Winn *et al.*, 2011) was used to verify the space group and to improve the model. Refinement was performed with BUSTER (Bricogne *et al.*, 2011) alternated with manual rebuilding in O (Jones *et al.*, 1991). Solvent molecules were added using the water insertion command in BUSTER. Assignment of water molecules was made based on peak heights of residual electron density, hydrogen-bonding patterns, and B-factors. Analysis of ligand occupancy was performed with Phenix (Adams *et al.*, 2010). Data collection and refinement statistics are presented in Table 1.

**Table 1.** Data collection and refinement statistics for *Se*CcmP Values in parentheses are for the outer resolution shell.

	*Se*CcmP_*P*2 _ 1 _ 3	*Se*CcmP_*I*2 _ 1 _ 3
Protein Data Bank id	5LT5	5LSR
**Data collection**		
Beam line	ID29	ID29
Wavelength (Å)	0.97239	0.97625
Space group	*P*2_1_3	*I*2_1_3
Unit cell parameters (Å)	*a*, *b*, *c=*107.2; α, β, γ=90°	*a*, *b*, *c=*178.7; α, β, γ=90°
*V*_M_ (Å^3^ Da^–1^)	2.1	3.4
Solvent content (%)	42	64
Resolution (Å)	47.9–1.45 (1.47–1.45)	44.7–1.65 (1.68–1.65)
No. of observations	721 918 (34 433)	2 272 675 (98 426)
No. of unique reflections	72 625 (3579)	113 271 (5592)
*R*_meas_^*a*^	0.061 (0.943)	0.095 (1.445)
<I/(σI)>	21.7 (2.6)	18.9 (2.3)
Completeness (%)	100.0 (100.0)	100.0 (100.0)
Multiplicity	9.9 (9.6)	20.1 (17.6)
**Refinement**		
Resolution range (Å)	47.9–1.45 (1.49–1.45)	44.7–1.65 (1.69–1.65)
No. of reflections	72 565	113 268
*R*_work_^*b*^	0.165	0.181
*R*_free_^*c*^	0.184	0.191
No. of atoms		
Protein	3246	4774
Ligands	13	6
Waters	325	357
Average *B*-values (Å^2^)		
Estimated from Wilson plot	19.2	29.2
Protein	20.1	32.1
Ligands	26.1	37.2
Waters	33.1	39.8
Rms deviations from ideal values		
Bond lengths (Å)	0.010	0.010
Bond angles (°)	1.05	1.03
Ramachandran analysis^*d*^		
Outliers (%)	0	0

^*a*^ *R*_meas_=∑_*h*_ ∑_*l*_ (n_*h*_/n_*h*_−1)^1/2^|*I*_*hl*_−<*I*_*h*_>|/∑_*h*_ ∑_*l*_<*I*_*h*_> (Evans, 2006; Evans and Murshudov, 2013).

^*b*^ *R*_work_=∑_*hkl*_||*F*_o_|−|*F*_c_||/∑_*hkl*_ |*F*_o_| where *F*_o_ and *F*_c_ are the observed and calculated structure factor amplitudes, respectively.

^c^ *R*_free_ calculated from a randomly chosen 5% of all unique reflections.

^*d*^From *MolProbity* (Chen *et al.*, 2010).

### Structure analysis

The structures were evaluated using wwPDB Validation Server (Berman *et al.*, 2003). Electron density fit was analysed using Coot (Emsley *et al.*, 2010). The DALI server (Holm and Rosenström, 2010) was used to identify similar structures in the PDB, and the lsq commands in *O* (Jones *et al.*, 1991) were used for comparison of *Se*CcmP_*P*2_1_3 chain B with hits in the DALI server.

The figures were created with *PyMOL* (Version 1.6.0.0, Schrödinger, LLC).

## Results

### Structure solution

*Se*CcmP was expressed in *E. coli* with a C-terminal 6×histidine-tag. Crystallization could only be achieved at a relatively low protein concentration (3 mg ml^−1^) and using freshly purified *Se*CcmP because protein precipitation occurred at higher concentration and lower (cold room) temperatures. Crystals appeared after incubation at 293 K for a few days and belong to the cubic space group *P*2_1_3 with two monomers per asymmetric unit. The phases were obtained using the molecular replacement method. Data to 1.45 Å resolution were included in the processing and refinement of the final model, *Se*CcmP_*P*2_1_3. When glycerol was omitted from the purification and crystallization protocols, *Se*CcmP crystallized in space group *I*2_1_3 with three molecules per asymmetric unit. The resulting model was refined to 1.65 Å resolution and is referred to as *Se*CcmP_*I*2_1_3. Statistics for data collection and refinement are presented in Table 1.

### Overall structures

**Fig. 1.**
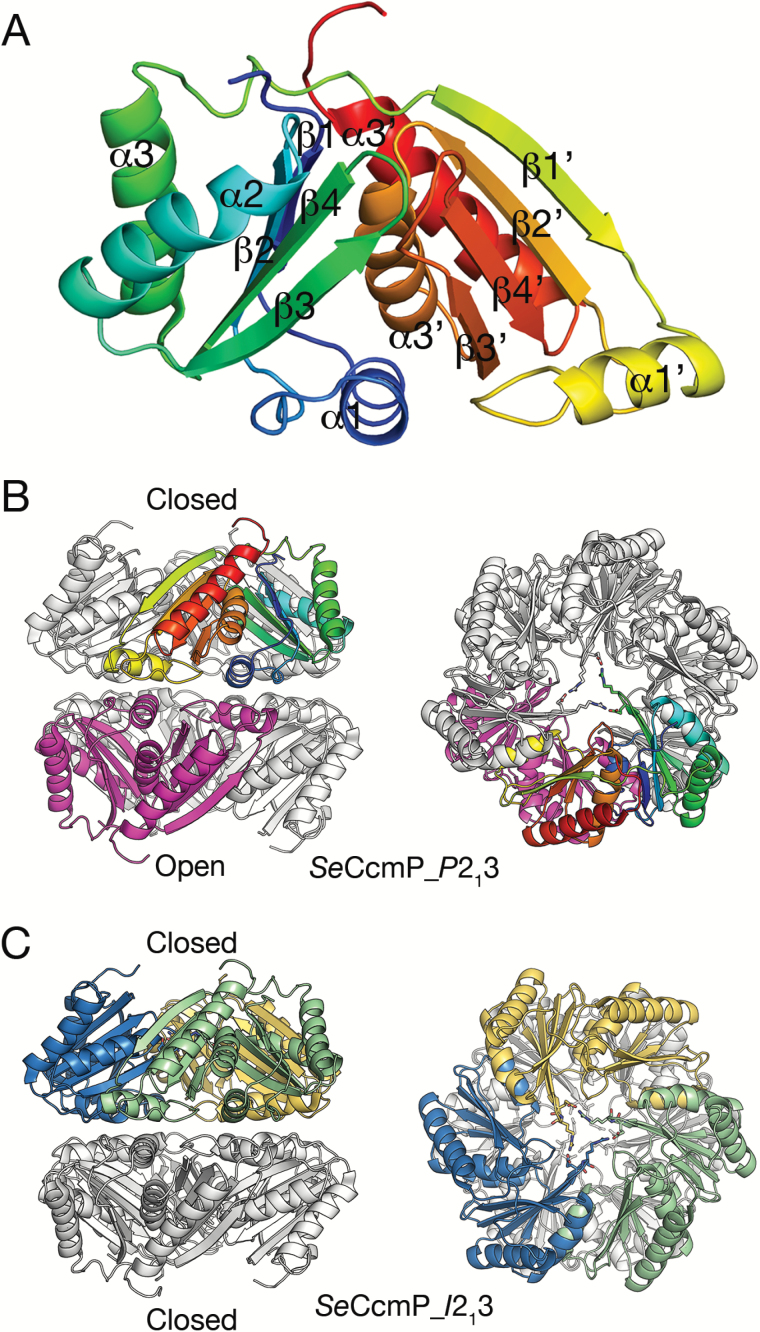
Overall structure of *Se*CcmP and arrangement of the six monomers in the asymmetric unit. (A) The *Se*CcmP monomer, consisting of two BMC domains, rainbow coloured from blue at the N-terminus to red at the C-terminus. (B) The *Se*CcmP_*P*2_1_3 structure with two monomers per asymmetric unit. One monomer (rainbow coloured) has the gating residues in a closed conformation, the second monomer (magenta) has the gating residues disordered. The biologically relevant hexamer is generated by adding symmetry-related monomers (grey). (C) The *Se*CcmP_*I*2_1_3 structure with three molecules per asymmetric unit (blue, yellow, and green) and the gating residues in a closed conformation. Crystal symmetry-related monomers in the biologically relevant hexamer are coloured grey. The two views in (B) and (C) are related by a 90 ° rotation with respect to the horizontal axis.

The *Se*CcmP monomer contains two BMC domains in tandem, the N- and C-BMC domains, each consisting of an α+β fold with three α-helices and a single β-sheet formed by four antiparallel β-strands (Fig. 1A). The *Se*CcmP_*P*2_1_3 structure contains two polypeptide chains in the asymmetric unit, chain A with 204 and chain B with 205 amino acids out of a total of 213 (residues Val3 or Gly2, respectively, to His206). In addition we identified 325 water molecules, two glycerol molecules, and a chloride ion, which is located between two hexamers in the crystal lattice. Seven amino acids in the C-terminus of *Se*CcmP, preceding the histidine-tag in the construct, were disordered and could not be modelled in the structure because of lack of interpretable electron density. The *Se*CcmP_*I*2_1_3 structure contains three monomers per asymmetric unit consisting of amino acids Val3 (chains A and C) or Gly2 (chain B) to Pro207. The stereochemistry of *Se*CcmP_*P*2_1_3 and *Se*CcmP_*I*2_1_3 is excellent, with no Ramachandran outliers and 99% and 98%, respectively, of the residues in the most favoured region. Two residues (Gly69 and Tyr72) in *Se*CcmP_*P*2_1_3 adopted more unusual conformations (identified as non-rotamer side chains in the wwPDB validation report; Berman *et al.*, 2003). Tyr72 in chain A is located in a flexible region and displays weak density. Glu69 in chain B has well-defined density and is one of the two principal pore-closing residues (see below). In the *Se*CcmP_*I*2_1_3 structure, Glu69 and Arg129 adopt unusual (non-rotamer) side chain conformations in all three monomers of the asymmetric unit.

The overall *Se*CcmP quaternary structure is a barrel made up by six *Se*CcmP monomers in two trimeric rings stacked on top of each other, with the same faces interacting (Fig. 1B). The two ends of the barrel feature pores that may be either open or closed depending on the conformations of residues lining the apertures at the three-fold axis. In particular, two highly conserved residues, Glu69 and Arg70, adopt different conformations in the two monomers in the asymmetric unit of the *Se*CcmP_*P*2_1_3 structure (Fig. 1B), resulting in a shift of the polypeptide backbone around these gating residues. In the *Se*CcmP_*P*2_1_3 structure, one monomer (chain A) forms a trimeric ring with an open central pore on the three-fold symmetry axis, whereas the second monomer (chain B) forms a ring with a closed central pore. This arrangement of the two rings leads to formation of a barrel with one end open and the other closed. The three monomers in the *Se*CcmP_*I*2_1_3 structure form a ring with a closed end. Addition of a second trimeric ring related by crystal symmetry results in a barrel with both ends closed (Fig. 1C).

In several places, additional electron density was observed that could not be assigned to protein. These densities occurred reproducibly in multiple structures, but unambiguous interpretation was not possible in most cases. The difficulties in interpreting the extra electron density might be explained by unspecific binding and the propensity of CcmP to interact with other shell proteins.

### Open and closed conformations

**Fig. 2.**
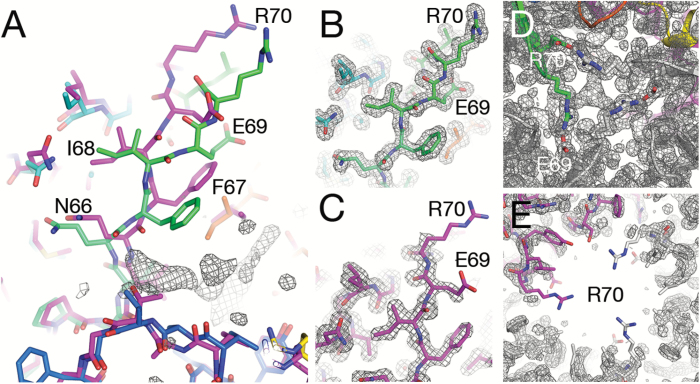
Gating residues Glu69 and Arg70 in open and closed conformations in the two monomers of *Se*CcmP_*P*2_1_3. (A) Superposition of the two monomers of *Se*CcmP_*P*2_1_3. Monomer A (magenta) in an open conformation and monomer B (rainbow coloured) in a closed conformation. The electron density of the ligand extends from the binding pocket of monomer B toward the carbonyl oxygen of Gln66. (B) Well-defined electron density for the gating residues in the closed conformation (monomer B). (C) Electron density for the gating residues in the open conformation (monomer A). (D) The closed end of the pore seen down the three-fold symmetry axis. (E) The open end of the pore seen down the three-fold symmetry axis. Electron densities are from 2*F*_o_–*F*_c_ maps contoured at the 1 σ level.

The two molecules in the *Se*CcmP_*P*2_1_3 structure that represent the open and closed conformations superimpose well overall (rms deviations of 0.739 Å for 203 equivalent Cα atoms out of a total of 204 amino acids). This superposition uses the default cut-off limit for equivalent Cα atoms of 3.8 Å; as a result, Arg70 is the only Cα atom that does not fall within the cut-off limit. In fact, the largest differences between the open and closed conformations of *Se*CcmP_*P*2_1_3 are observed for residues 66–72 in a β-hairpin structure comprising the gating residue Arg70 (Gln66 1.4 Å, Phe67 1.5 Å, Ile68 1.1 Å, Glu69 3.4 Å, Arg70 5.4 Å, Leu71 3.0 Å, and Tyr72 1.4 Å). In the closed state, there is an interaction between Glu69 and Arg70 from two adjacent protein molecules (Fig. 1B), which effectively closes the pore. In the transition from the closed to open state, the side chain of Arg70 changes its conformation and interacts with Glu69 in the monomer on the opposite side. Despite the large movement of Arg70 (>12 Å movement of its guanidinum group), the structure of the β-hairpin is essentially intact and shifted such that the hydrogen bonds between β-strands β3 and β4 including the interactions between Glu69 and Tyr72 and between Phe67 and Leu74 are kept intact. Besides the changes in position of the gating residues Glu69 and Arg70, the side chain of Phe67 displays a large positional shift (Fig. 2A). Weaker electron density is observed for residues 67–72 in the open conformation compared with the corresponding residues in the closed conformation, indicating a higher flexibility of this region for the open state (Fig. 2B–E). Most notable is the lack of density for the carboxyl group of Glu69 and nearly all of the side chain of Arg70 in monomer A, which forms the open pore. The same tendency, but with only slightly lower density fit for the open state, is observed in loop A171–A175 of the C-BMC domain, which corresponds to the N-BMC loop containing Glu69 and Arg70. The side chain of Ile172 assumes different conformations in the two states; this shift appears to be coupled to the large displacement of Phe67, with which it forms hydrophobic interactions.

The three monomers in *Se*CcmP_*I*2_1_3 form a pseudohexameric ring with a closed pore (Fig. 1C). Crystallographic symmetry-related monomers make up the second ring, resulting in a barrel that is closed at both ends. The three molecules in the structure superimpose with low rms deviations (0.122–0.140 Å) when including 205 Cα atoms. Superposition of the two different structures shows that *Se*CcmP_*I*2_1_3 (chain A) is most similar to the closed conformation chain of *Se*CcmP_*P*2_1_3 (chain B, rms deviation 0.333 Å). The open conformation *Se*CcmP_*P*2_1_3 chain A superimposes on *Se*CcmP_*I*2_1_3 (chain A) with an rms deviation of 0.701 Å.

### Binding pocket at the interface of the N- and C-BMC domains

**Fig. 3.**
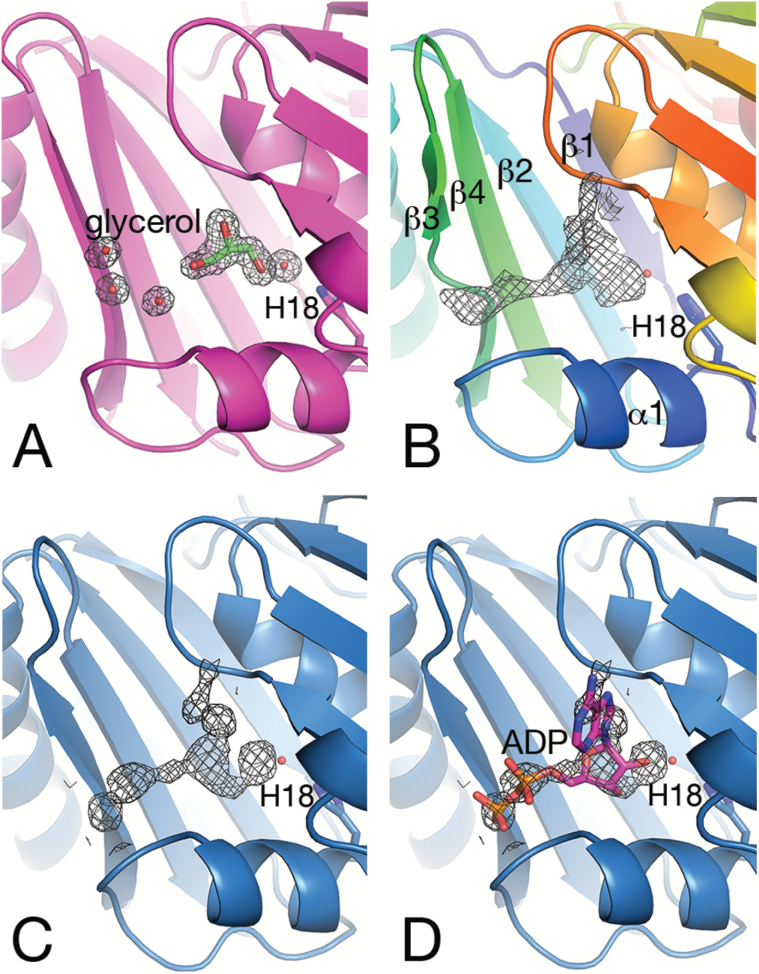
Electron density 2*F*_o_–*F*_c_ maps in the binding pocket contoured at the 1 σ level. The side chain of His18 and the adjacent water molecule are shown as a stick representation and a red sphere, respectively. (A) Monomer A of *Se*CcmP_*P*2_1_3 (magenta) with the gating residues in an open conformation binds glycerol and a water molecule in the binding pocket between the N- and C-BMC domains. (B) Monomer B of *Se*CcmP_*P*2_1_3 (rainbow coloured from blue in the N-terminus to red in the C-terminus) with an extended electron density and the gating residues in a closed conformation. (C) Monomer A of *Se*CcmP_*I*2_1_3 (blue) with the gating residues in a closed conformation displaying a similar density as in (B). (D) ADP modelled into the density in (C).

*Se*CcmP contains a binding pocket which is located between the N- and C-BMC domains (Cai *et al.*, 2013). The hexameric structure of *Se*CcmP thus potentially gives rise to six binding pockets that line the interior of the barrel. The pocket is confined by residues from helix α1 and strand β3 from the N-BMC domain and strand β3' of the C-BMC domain. In particular, a conserved histidine residue (His18) is strategically located in the innermost part of the binding pocket. After careful refinement of the protein, residual electron density was apparent in the pockets. The shape of the density showed distinct differences depending on the conformation, open or closed, of the *Se*CcmP molecule. Well-defined electron density for solvent molecules (glycerol and a water molecule) was observed in the pocket of the *Se*CcmP_*P*2_1_3 monomer forming the open pore (Fig. 3A). The water molecule is at hydrogen-bonding distance to the ε^2^-nitrogen atom of His18 and one of the hydroxyl oxygen atoms of glycerol. In the pocket of the second molecule in the asymmetric unit, with the gating loop in a closed conformation, the interpretation of the electron density was more ambiguous (Fig. 3B). Electron density potentially originating from a water molecule is situated 2.8 Å from the ε^2^-nitrogen of the His18 imidazole ring in a position corresponding to the location of the solvent molecule in the pocket of the open pore molecule. Adjacent to this solvent site, an elongated electron density was observed that expands in two directions (Fig. 3B). The density is bounded on one side by the backbone carbonyl group of Val169 and the backbone amide nitrogen atoms of Ala171 and Ile172. Extending toward the N-BMC domain, a strong patch of electron density is positioned 3.6 Å from the backbone amide nitrogen of Gln66 in the β-strand preceding the loop that determines the open or closed state of the pore (see below; Fig. 2A). The peak is located 10 Å away from the water molecule that interacts with His18. This is a reasonable distance between the two phosphate groups of the substrate RuBP (Duff *et al.*, 2000) which was present during crystallization. Considering that the density is strongest at the centre, the binding of a mixture of compounds in the closed monomers B of the CcmP_*P*2_1_3 crystal may be considered, which manifests itself as a complex and smeared density in the electron density maps.

The discovery of well-defined density for a glycerol molecule in the binding pocket of the open-pore monomer prompted a revision of the purification and crystallization protocols omitting glycerol. The resulting protein crystallized in space group *I*2_1_3 in the presence of thiocyanate and assumed a closed conformation only. Elongated electron densities of a similar shape were also observed in all three binding pockets of the closed conformation *Se*CcmP_*I*2_1_3 structure (Fig. 3C). In monomer A, the density displays two strong peaks 2.8 Å apart, with the more distant peak 3.3 Å away from the backbone amide nitrogen of Gln66. The electron density in the cleft is not a chance observation; similarly shaped densities were consistently observed in all data sets analysed in this project (unpublished results). Co-crystallization and soaking experiments in order to elucidate the identity of the bound ligands have so far been inconclusive. Bicarbonate and 3PGA used in co-crystallization experiments were not detected and may have been outcompeted by the cryoprotectant.

Model building of RuBP into the difference density did not give a conclusive result. Given the shape of the density (resembling a boomerang), we identified ADP as a possible candidate to fit the density, with the ribose ring at hydrogen-bonding distance to the water molecule, the two phosphate groups pointing toward the N-BMC domain, and the adenine ring contacting strand β3 of the C-BMC domain. Refinement of the structure with ADP present was not convincing, and the estimated occupancy of ADP was low (0.3–0.4). Weak density was present for the adenine ring, and the positioning of ADP resulted in too short contact distances to the protein in several places. However, some of the residues close to the ligand have dual conformations, indicating the presence of two subpopulations. Co-crystallization of ADP or ATP increased the occupancy slightly, but did not result in well-defined density for the ligand (unpublished results). Considering these uncertainties, we have opted not to include a ligand in the final model and conclude that the binding of a larger molecule, extending toward Thr27 and the backbone nitrogen of Gln66, coincides with the gating residues in a closed conformation, whereas the binding of a smaller molecule, corresponding to glycerol present in one of our crystallization conditions, coincides with an open pore.

### Structural comparison with other BMC shell proteins

Two structures of *Se*CcmP are available in the PDB—a 2.51 Å resolution structure in space group *P*2_1_2_1_2 and a low-resolution (3.3 Å) structure in space group *P*2_1_2_1_2_1_ (PDB codes 4HT5 and 4HT7, respectively; Cai *et al.*, 2013). In the former structure, a solvent content of 70.7% and empty space between layers of well-defined *Se*CcmP hexamers indicate that not all molecules in the asymmetric unit had been located. The difficulties in placing molecules in the missing layer were suggested (Cai *et al.*, 2013) to be caused by lattice-translocation disorder, as previously detected in CsoS1C (Tsai *et al.*, 2009), but no analysis of native Patterson peaks or local intensity differences were presented to justify this proposal and no corrections were applied. The high *R* and *R*_free_ factors for the *P*2_1_2_1_2 structure may thus be a result of parts missing from the model, causing problems during refinement. The 3.3 Å resolution *Se*CcmP structure provides limited information due to the low resolution. In addition, two structures of the related CsoS1D from the α-carboxysome *Prochlorococcus marinus* subsp. *pastoris* str. CCMP1986 (Klein *et al.*, 2009) are available in PDB; a well-refined SeMet-substituted structure to 2.3 Å resolution (PDB code 3F56) and a native structure to 2.2 Å resolution, which was solved from merohedrally twinned data with a twin factor of 0.45 and a very low fit (23.2% RSRZ outliers) to the density (PDB code 3FCH).

When comparing a single BMC domain (residues 3–103 or 104–206) of *Se*CcmP with other structures in the PDB using DALI, the more similar structure unexpectedly appears to be the SeMet-substituted structure of CsoS1D (Klein *et al.*, 2009), and not the previously deposited *Se*CcmP structures (Cai *et al.*, 2013). The same result is obtained using either the N-BMC or C-BMC domains as search models. The higher *Z*-score may be a result of both CsoS1D and *Se*CcmP_*P*2_1_3 being well-refined structures and may be less influenced by the lower sequence identity (48% compared with identical) in this case.

If the complete two-domain *Se*CcmP_*P*2_1_3 structure is used in the DALI search, *Se*CcmP structures are given the highest similarity scores followed by the *P. marinus* CsoS1D structures (Table 2). Surprisingly, the next structures in the list are PduT from *Citrobacter freundii* and a PduT analogue from *Desulfitbacterium hafniense*. PduT is a shell protein with tandem repeats. The two domains are not circularly permuted in this case, but feature a duplication of a canonical domain. A lower similarity score is obtained for PduT compared with the other proteins if single domains are used for the comparision. Slightly lower *Z*-scores were observed for PduU and EutS (9.2 and 10.2, respectively); these are shell proteins that contain a single permuted BMC domain. Despite higher sequence identity, shell proteins with permuted tandem repeats get a lower *Z*-score, when the complete structures are compared, than the permuted single domain proteins (Table 2). The higher similarity to PduT when using the complete *Se*CcmP_*P*2_1_3 molecule as a search model indicates that the two domains of *Se*CcmP_*P*2_1_3 are arranged in a way more similar to PduT than to the other non-carboxysomal permuted tandem proteins.

**Table 2.** Structure comparison of *Se*CcmP_*P*2_1_3 chain B (closed) with BMC shell proteins (permuted and/or duplicated) in the PDB

Protein	Organism	PDB	No. of aa^*a*^	*Z*-score	Rmsd	No.^*b*^	No. id^*c*^
CcmP	*Synechococcus elongatus* PCC 7942	4HT5-A^*d*^	204	39.1	0.252	204	204
CsoS1D	*Prochlorococcus marinus* MED4	3F56-A^*e*^	207	34.8	0.667	201	91
PduT	*Citrobacter freundii*	3PAC-A^*f*^	183	11.1	1.608	90	19
EutS	*Escherichia coli* K-12	3IA0-E^*g*^	111	10.2	1.236	88	17
PduU	*Salmonella enterica*	3CGI-C^*h*^	119	9.2	1.271	82	17
EutL	*Clostridium perfringens*	4U6I-C^*i*^	216	8.5	1.673	157	32
EtuB	*Clostridium kluyveri* DSM 555	3IO0-A^*j*^	229	8.1	1.624	82	13
GrpU	*Clostridiales bacterium* 1_7_47_FAA	4OLO-B^*k*^	84	6.9	1.380	74	12
PduB	*Lactobacillus reuteri* SD2112	4I61-C^*l*^	227	6.4	1.536	76	12

^*a*^No. of aa, number of amino acids in the chain.

^*b*^No., number of amino acids within the 3.8 Å cut-off.

^*c*^No. id, number of identical residues within the cut-off.

^*d*^Cai *et al.*(2013).

^*e*^Klein *et al.* (2009).

^*f*^ Pang *et al.* (2011).

^*g*^Tanaka *et al.* (2010).

^*h*^Crowley *et al.* (2008).

^*i*^Thompson *et al.* (2014*a*).

^*j*^Heldt *et al.* (2009).

^*k*^Thompson *et al.* (2014*b*).

^*l*^Not published.

### Communication between binding sites

**Fig. 4.**
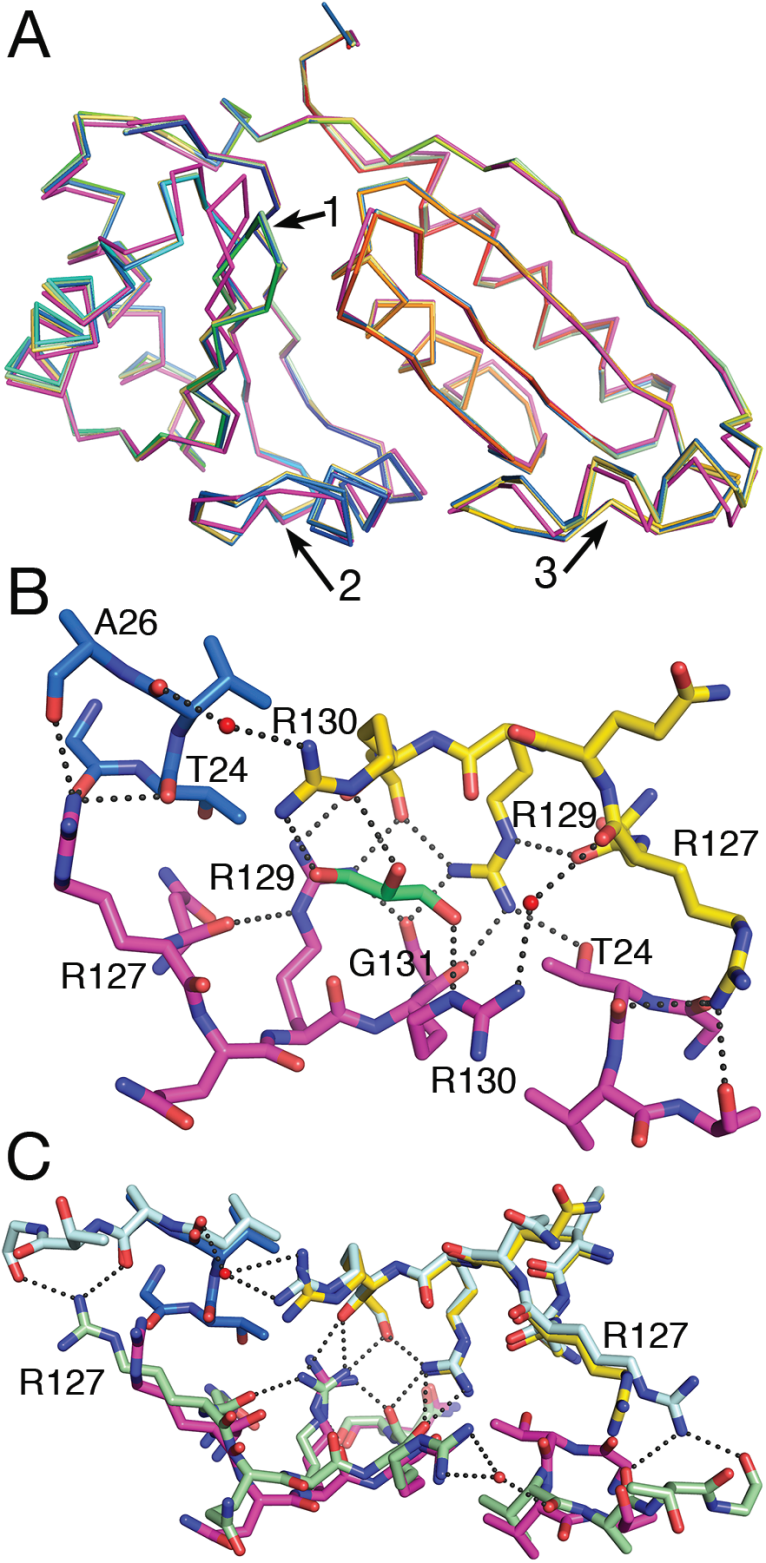
Interactions between monomers in the CcmP hexamer. (A) Differences in the *Se*CcmP main chain backbone in the open and closed conformation shown by superimposing *Se*CcmP_*P*2_1_3 monomer A (magenta) and monomer B (rainbow) and *Se*CcmP_*I*2_1_3 monomer B (yellow) and monomer C (green) on *Se*CcmP_*I*2_1_3 monomer A (blue). The only monomer with the gating residues in an open conformation is *Se*CcmP_*P*2_1_3 chain A (magenta). Structural deviations of the backbone in open and closed conformations are observed in the gating loops region (arrow 1), in helix α1 and the following loop region of the N-BMC domain (arrow 2) and in the corresponding helix α1'-loop region in the C-BMC domain (arrow 3). (B) Interactions in the helix–loop region between the two molecules in the asymmetric unit of *Se*CcmP_*P*2_1_3 (monomer A magenta and monomer B rainbow coloured). Salt bridges between Arg127 and Arg129 to backbone carbonyl groups and solvent form a rigid network of interactions that may mediate changes in the binding site of one trimeric ring to the binding site in the second ring. (C) *Se*CcmP_*I*2_1_3 monomer A (light blue) superimposed on *Se*CcmP_*P*2_1_3 chain B (rainbow coloured), and *Se*CcmP_*I*2_1_3 monomer C (green) superimposed on *Se*CcmP_*P*2_1_3 monomer A (magenta). Note the differences in salt bridges formed by the side chains of Arg127 in the two structures.

To date, three out of four available structures of the tandem BMC domain proteins CcmP and CsoS1D feature open and closed pores in the same barrel (Klein *et al.*, 2009; Cai *et al.*, 2013). This may suggest communication between the open and closed states such that one end of the barrel is closed (gating residues organized), while the other end is open (gating residues flexible). Such gating may be controlled by the binding of an extended molecule in one site making the binding in the other site less likely. To elucidate such interactions, we superposed *Se*CcmP_*P*2_1_3 chains A and B and *Se*CcmP_*I*2_1_3 chains B and C on *Se*CcmP_*I*2_1_3 chain A (Fig. 4A). The strongest electron density peaks of the potential ligand are positioned within contact distance to the main chain carbonyl group of Gln66 in strand β3 and the side chain of Thr27 in the loop following helix α1 in the N-BMC domain. The largest structural differences between the monomers that make up the open and closed conformers are localized to the gating loop containing Glu69 and Arg70, but we also observe distinct differences in helix α1 in the N-BMC domain and in the corresponding helix α1'-loop region of the C-BMC domain. The helix α1-loop region of the N-BMC domain of monomer B, featuring a closed central pore, interacts with Arg127 of the helix α1'-loop of the C-BMC domain in the trimeric ring with an open central pore (monomer A) via three hydrogen bonds to the backbone carbonyl groups of Gly23, Thr24, and Ala26. Arg127 in *Se*CcmP_*I*2_1_3 adopts a more extended conformation, compared with in *Se*CcmP_*P*2_1_3, and forms hydrogen bonds to the carbonyl groups of Ala26 and Gly28 (Fig. 4C). The two C-terminal helix α1-loop regions from the open and closed monomers are interacting symmetrically with each other via several hydrogen bond interactions between Arg129 in chains A/B and the backbone carbonyl groups of Arg130 and Gly131 in chains B/A (Fig. 4B). Arg129 also forms a hydrogen bond to the carbonyl group of Gly131 in the same polypeptide chain; this contact further stabilizes the interactions between the two helix–loop regions of the C-BMC domains. Thus Arg127 of the closed pore trimer may affect the helix–loop region of the open pore trimer through this network of hydrogen bonds.

In the CsoS1D and CcmP structures (Klein *et al.*, 2009; Cai *et al.*, 2013), which display open and closed conformations in the same barrel, Arg127, or the corresponding Arg178 in CsoS1D, is in an extended conformation in the open pore trimer and in a bent conformation in the closed pore trimer. The conflicting result seen for *Se*CcmP_*P*2_1_3 and *Se*CcmP_*I*2_1_3, where Arg127 is in an extended conformation in the closed/closed structure and in a bent conformation for the open/closed structure, may be explained by high concentration of molecules that bind to the protein during crystallization (Fig. 4C). In *Se*CcmP_*P*2_1_3, density corresponding to a glycerol molecule is present at the interface forming hydrogen bonds to Arg130 in both monomers A and B (Fig. 4B). *Se*CcmP_*I*2_1_3 crystallizes in the presence of thiocyanate. Densities interpreted as thiocyanate were observed close to Phe29 in monomers A and C. The binding of thiocyanate may impair Phe29 from assuming the two different conformations observed in the open and closed conformations of *Se*CcmP_*P*2_1_3.

## Discussion

Structure determination of BMC shell proteins has proved to be a challenging task (Klein *et al.*, 2009; Tsai *et al.*, 2009; Cai *et al.*, 2013; Thompson and Yeates, 2014). Non-crystallographic symmetry may give rise to twinning, and preference for packing in layers can result in disorder of entire layers and the inability to trace all the molecules present in the asymmetric unit. This has resulted in difficulties in refining the structures, yielding high *R* factors. In this context, our well-refined high-resolution *Se*CcmP structures add important information and provide models for further analysis.

Speculation that some carboxysomal shell proteins function as selective channels with gated pores to allow the passage of substrate or cofactors through the microcompartment shell was inspired by the three-dimensional structure of CsoS1D—the CcmP orthologue in α-carboxysomes (Klein *et al.*, 2009). In the crystal structure of CsoS1D, one trimer features a pore that is closed by the formation of salt bridges between conserved glutamate and arginine residues, whereas the second trimer of the hexamer has a relatively large open pore. Both these two-domain BMC proteins feature circularly permuted secondary structure elements not observed in the more abundant single-domain shell proteins in both types of carboxysomes.

Recently, molecular transport through the protein shell of another type of microcompartment, the metabolosome, was suggested. Chowdhury *et al.* (2015) described how PduA may selectively transport the substrate 1,2-propanediol into the Pdu microcompartment while the outflow of the toxic intermediate propionaldehyde is restricted. The trimeric PduB is another pseudohexameric shell protein suggested to be a selective transporter of glycerol (Pang *et al.*, 2012), a second substrate metabolized in the Pdu microcompartment. In the PduB crystal structure (PDB code 4FAY) glycerol molecules are bound to conserved residues in the three channels formed between the two BMC domains within each PduB subunit (Pang *et al.*, 2012). For the related protein EutL in the ethanolamine utilization (Eut) microcompartment, structures are available with a closed pore (PDB code 3I82), as in the PduB structures, but also with an open 10–12 Å wide central pore (PDB code 3I87) on the three-fold symmetry axis of the trimer (Tanaka *et al.*, 2010). The binding of the substrate, ethanolamine, in the three channels within each subunit was suggested to be a negative allosteric control that prevents the open conformation of the pore (Thompson *et al.*, 2015).

The binding pocket between the two BMC domains of *Se*CcmP contains a conserved His18. Conservation is not strict between α- and β-carboxysomes; in CsoS1D, the corresponding residue is a leucine. The difference electron density map for the CsoS1D structure showed additional density near the modelled water molecules in the regions corresponding to the binding pocket. The additional density extends from SeMet73 toward the opening of the cleft (selenomethionine replaces methionine in CsoS1D to facilitate phasing; Klein *et al.*, 2009) and indicates that a molecule larger than water is bound in the pocket. Alternatively the density might just be an artefact due to the incorporation of a selenium atom instead of a sulphur atom in residue 73.

No residue with a similar function to His18 in *Se*CcmP could be identified in the corresponding cleft of Csos1D. However, on the other side of strand β3 and the gating loop in the CsoS1D structure, His100—conserved in α-carboxysomes from both cyanobacteria and chemoautotrophs (Cai *et al.*, 2013)—is located in a pocket where a molecule may bind and stabilize the closing of the pore. Additional density was observed in *Se*CcmP_*I*2_1_3 and *Se*CcmP_*P*2_1_3 monomer B (closed conformation) in the cleft that corresponds to the His100 cleft in CsoS1D, but not in *Se*CcmP_*P*2_1_3 monomer A (open conformation). Differences in the binding pockets of the α- and β-carboxysomes may be a result of distinct preferences for the molecule bound. Alternatively, and perhaps more likely, the gating control has developed independently from a common ancestor.

The *Se*CcmP_*P*2_1_3 structure to 1.45 Å resolution presented herein reveals that glycerol was bound in the binding pocket between the N- and C-BMC domains close to the gating loop when the pore is open, whereas a density for a larger molecule was observed in the binding pocket when the pore is closed. In earlier structures of *Se*CcmP, Cai *et al.* (2013) observed density corresponding to a 2–4-carbon molecule in all six binding pockets, and interpreted this as 3PGA included in the crystallization solution (Cai *et al.*, 2013). Based on this scattered information, an attractive model emerges for the gated transport through the CcmP shell, in which the binding of the shorter product 3PGA promotes the opening of the pore while the binding of the larger substrate RuBP promotes the closing. This model is only partly supported by our structural data, which show the binding of glycerol in the pocket of the open pore monomer, whereas a larger, as yet unidentified, molecule binds in the pocket of the closed pore monomer of *Se*CcmP. Our co-crystallization and soaking experiments aimed at elucidating the identity of the bound compounds have so far been inconclusive. The smaller HCO_3_^−^ and 3PGA were not observed and either may have been outcompeted by cryoprotectants or may not have bound. The co-crystallization or the soaking into pre-formed crystals of the larger compounds, RuBP and ADP/ATP, did not result in well-defined density. There may be several reasons for this. Binding may be hindered by the crystal lattice packing. Alternatively, the binding of a metabolite in the pocket during protein expression and purification may have blocked the binding of other compounds during crystallization. However, the high concentration of RuBP (50 mM) used during crystallization makes this unlikely, at least if this site is poised to bind RuBP. It is also possible that the crystal lattice may have forced the protein into a slightly altered conformation that does not permit the binding of the compound. The low occupancy may also be a result of the protein allowing the binding of the ligand in only one out of the three pockets, which would then be localized randomly within the trimer in the crystal. Given the proposed nature of CcmP as a carrier of metabolites in and out of the carboxysome shell, weak binding and low occupancy of the ligand is to be expected. Finally, we note that there may be an advantage of using a metabolite related to the energy content of the cell as a gating signal.

The answer to the nature of the conformational switch clearly awaits further research, but we note the following: β-carboxysomes have not yet been purified to homogeneity and it is not known if ADP is able to enter the carboxysome. However, the occurrence in some organisms (albeit not in *S. elongatus*) of genes for the ATP-dependent Rubisco activases, Rca, CbbX, and CbbQ, with their carboxysome operon (Zarzycki *et al.*, 2013; Sutter *et al.*, 2015) hints at the targeting of these proteins to the carboxysome and indicates that the possible binding of ADP to CcmP may not be as far-fetched as may at first seem. According to the taxonomy classification by Axen *et al.* (2014), some metabolosome genetic loci with unknown function are described to have an incomplete core, with enzymes responsible for cofactor recycling missing. Since one of the main functions of the characterized metabolosomes is believed to be the maintenance of a pool of recycled cofactors, the loss of the recycling machinery raises the question of the actual function of these types of metabolosome loci. For the type of metabolosomes where the machinery for recycling is not present, alternative solutions may be proposed; one of the suggestions is the selective transport of cofactors through the shells (Axen *et al.*, 2014). In the PDU microcompartment, the iron-dependent alcohol dehydrogenase (PduQ) is responsible for recycling of NAD^+^ (Cheng *et al.*, 2012), and a coenzyme A phosphotransacylase (PduL) recycles coenzyme A (Liu *et al.*, 2015). In the two loci of the type *Rhodococcus* and *Mycobacterium* Microcompartment (RMM), RMM1 and RMM2, the genes coding for the two enzymes responsible for recycling are missing from a set of core enzymes otherwise similar to the PDU loci (Axen *et al.*, 2014). Instead, the RMM loci contain genes coding for two CcmP homologues with the glutamate–arginine pair responsible for the pore closing conserved. The Metabolosomes with an Incomplete Core 1 (MIC1) locus has two putative alcohol dehydrogenase genes but no phosphotransacylase for the recycling of coenzyme A. A single gene for a CcmP homologue is also present in the MIC1 locus. It is tempting to speculate that these CcmP homologues function as gated pores for the selective transport of the cofactors through the BMC shell and that this may also apply to some carboxysomal shell proteins such as *Se*CcmP.

## Abbreviations:

BMC: bacterial microcompartment
PDB: Protein Data Bank
3PGA: 3-phosphoglycerate
Rubisco: ribulose-1,5-bisphosphate carboxylase/oxygenase
RuBP: ribulose 1,5-bisphosphate
*Se*CcmP_*I*2_1_3: *Synechococcus elongatus* PCC7942 crystallized in space group *I*2_1_3
*Se*CcmP_*P*2_1_3: *Synechococcus elongatus* PCC7942 crystallized in space group *P*2_1_3.
